# Rational Redesign of Monoamine Oxidase A into a Dehydrogenase to Probe ROS in Cardiac Aging

**DOI:** 10.1021/acschembio.0c00366

**Published:** 2020-06-26

**Authors:** Luca Giacinto Iacovino, Nicola Manzella, Jessica Resta, Maria Antonietta Vanoni, Laura Rotilio, Leonardo Pisani, Dale Edward Edmondson, Angelo Parini, Andrea Mattevi, Jeanne Mialet-Perez, Claudia Binda

**Affiliations:** † Department of Biology and Biotechnology, University of Pavia, Milan, Italy; ‡ Institute of Metabolic and Cardiovascular Diseases (I2MC), Institut National de la Santé et de la Recherche Médicale (INSERM), Université de Toulouse, Toulouse, France; § Department of Biosciences, University of Milan, Milan, Italy; ∥ Department of Pharmacy-Drug Sciences, University of Bari Aldo Moro, Bari, Italy; ⊥ Department of Biochemistry, Emory University, Atlanta, Georgia, United States

## Abstract

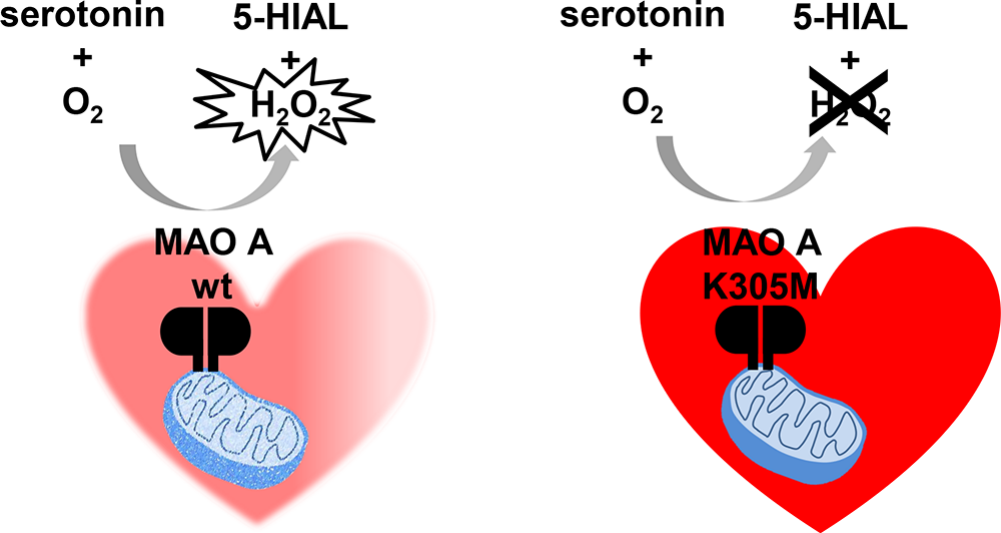

Cardiac senescence is a typical chronic frailty condition in the elderly population, and cellular aging is often associated with oxidative stress. The mitochondrial-membrane flavoenzyme monoamine oxidase A (MAO A) catalyzes the oxidative deamination of neurotransmitters, and its expression increases in aged hearts. We produced recombinant human MAO A variants at Lys305 that play a key role in O_2_ reactivity leading to H_2_O_2_ production. The K305Q variant is as active as the wild-type enzyme, whereas K305M and K305S have 200-fold and 100-fold lower *k*_cat_ values and similar *K*_m_. Under anaerobic conditions, K305M MAO A was normally reduced by substrate, whereas reoxidation by O_2_ was much slower but could be accomplished by quinone electron acceptors. When overexpressed in cardiomyoblasts by adenoviral vectors, the K305M variant showed enzymatic turnover similar to that of the wild-type but displayed decreased ROS levels and senescence markers. These results might translate into pharmacological treatments as MAO inhibitors may attenuate cardiomyocytes aging.

Oxidative stress is a pathophysiological condition resulting from an imbalance of cellular free radicals (reactive oxygen or nitrogen species, ROS or RNS, respectively) with respect to antioxidant defense systems.^1^ This process is associated with a number of diseases such as cancer and neurodegenerative disorders, though the underlying molecular mechanisms are not fully understood. Many pathological or frailty states may lead to oxidative stress, but in certain circumstances ROS generated by cell oxidative metabolism may contribute themselves to triggering a disease. In this context, the role of H_2_O_2_ is dual because, while being physiologically involved in many signaling pathways, it represents a deleterious source of ROS through the Fenton or Haber–Weiss reaction when produced in excess.^2^

Mitochondrial respiration is the main source of superoxide anions and H_2_O_2_, but the membrane of these organelles contains also other enzymes that generate ROS. Monoamine oxidases A and B (MAO A and MAO B, respectively) are 60-kDa mammalian flavoproteins that feature a water-soluble globular main body anchored to the outer mitochondrial membrane through a C-terminal α-helix.^3^ They belong to the FAD-dependent amine oxidase family of enzymes that catalyzes the oxidation of a carbon–nitrogen bond in various substrates with the concomitant reduction of the flavin coenzyme, which can be readily reoxidized by molecular oxygen leading to the generation of H_2_O_2_ (Figure 1).^4^ MAOs are particularly abundant in mammalian cells where they play a key role in the metabolism of both endogenous and exogenous neuroactive aromatic amines. The function of MAOs has been thoroughly studied in the central nervous system where they represent established drug targets for Parkinson’s disease and depression.^5^ However, MAOs are widely expressed also in non-neuronal tissues, including the heart, in which they regulate the local concentrations of serotonin, noradrenaline, and dopamine. Interestingly, MAO A levels in the heart increase significantly with aging.^6^ As a mimicry model of the elderly heart, the effects of cardiac overexpression and activation of MAO A in transgenic mice were studied by the authors of the present work, showing that an excess of H_2_O_2_ has multiple p53-mediated cell responses including mitochondrial damage and cell death.^7^ More recently, they also demonstrated that a chronic MAO A-dependent increase of H_2_O_2_ in heart cell lines triggers lipid peroxidation, elevated p53/p21 levels, DNA damage response, and other classical senescence markers.^8^ This cascade of events is associated with mitochondrial dysfunction, decreased respiration, and inhibition of parkin-mediated mitophagy. These findings have important clinical implications because MAO A-dependent release of H_2_O_2_ may occur either under acute pathological conditions such as ischemia-reperfusion injury when MAO substrates are heavily released or chronically when MAO A expression increases during aging.

**Figure 1:**
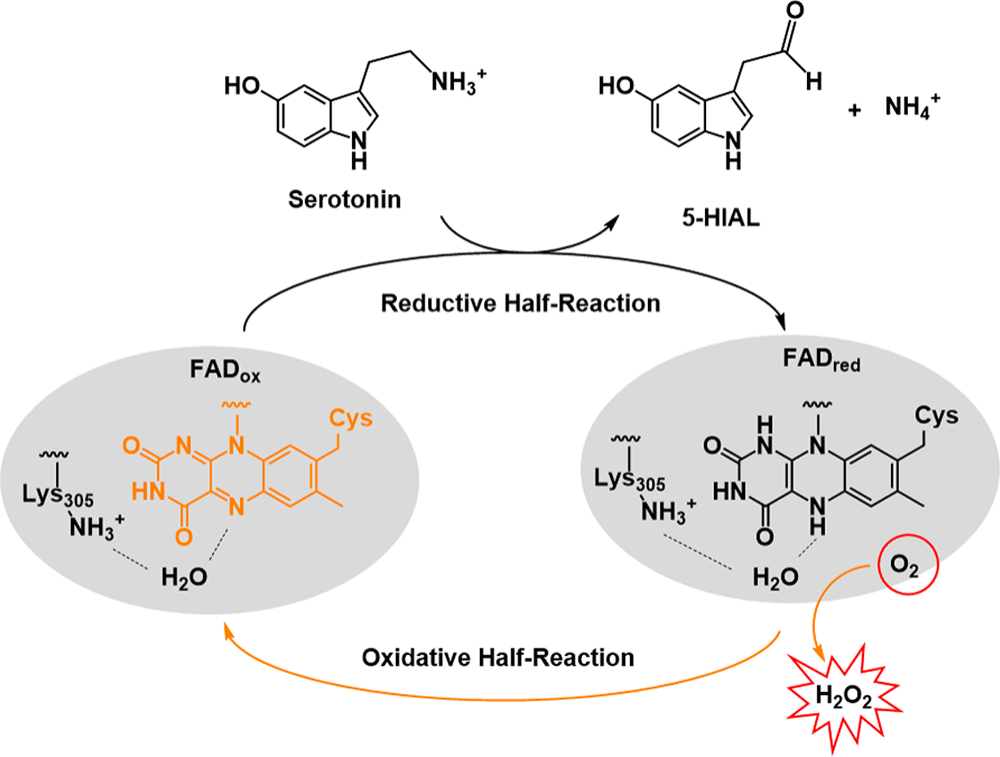
Scheme of amine substrate oxidation catalyzed by human MAO A (gray oval; the FAD coenzyme is covalently bound to a Cys residue). Oxidation of serotonin to 5-hydroxyindoleacetaldehyde (5-HIAL) is shown, with serotonin being the main MAO A substrate in heart metabolism. The two reductive and oxidative half reactions related to the flavin cofactor redox states are highlighted in black and yellow, respectively. In oxidases, the presence of a positive charge in proximity of the flavin isoalloxazine ring is believed to promote O_2_ binding and activation followed by H_2_O_2_ generation.^4^ In flavin-dependent amine oxidases, the conserved Lys305 lying on the top rim of the enzyme active site is H-bonded (dashed lines) to the flavin N5 atom through a bridging water molecule, which is believed to represent the O_2_ binding site.

Although we supported evidence for the involvement of H_2_O_2_ in acute and chronic cardiac damage, it is still uncertain whether MAO A-dependent substrate regulation may also have an impact on cardiac dysfunction. In an effort to probe the molecular features of human MAO A in relation to H_2_O_2_ production, we produced recombinant enzyme variants at the Lys305 site lying close to the flavin cofactor. Lys305 is a conserved residue that is engaged with the flavin ring through a hydrogen bond mediated by a water molecule (Figure 1).^3^ This site functions as a trap for molecular oxygen in flavin reoxidation following each MAO reductive half-reaction, which leads to H_2_O_2_ production.^9^ Mutation of this lysine residue was shown to abolish the catalytic activity of this class of amine oxidases,^10,11^ but the exact effects of this alteration were not studied in detail. In this work, we used Lys305 of human MAO A as a molecular tool to investigate the relevance of MAO A in age-related cardiovascular diseases based on the hypothesis that the increased enzyme-derived H_2_O_2_ levels may lead to oxidative stress. In particular, Lys305 mutants of human MAO A were tested through a combined enzymology/cell biology approach that involved activity assays on the purified proteins and overexpression of selected variants in the H9C2 cardiac cell line through an adenoviral vector. We show that the MAO A K305M mutant, which is seemingly inactive, is capable of oxidizing the amine substrate in the enzyme reductive half-reaction, while being impaired in enzymatic turnover due to lower reactivity with molecular oxygen. Nevertheless, its cellular enzymatic turnover can be restored by interaction with alternative electron acceptors without inducing the ROS-mediated senescence hallmarks of the wild-type enzyme.^8^

Four mutants of human MAO A (K305M, K305S, K305Q, K305R) were produced in *Pichia pastoris* as His-tagged recombinant proteins and purified as detergent preparations using protocols adapted from published procedures.^12^ These variants were designed on the basis of the interaction of Lys305 with the water molecule mediating the H-bond bridge with the reactive N5 atom of the flavin (Figure 1). First, we tested the enzymatic activity of the variants compared to wild-type enzyme by the horseradish peroxidase (HRP)-coupled assay using kynuramine as a substrate. This is a widely used method to measure the activity of oxidases that generate H_2_O_2_ in stoichiometric amounts with the product (see Methods in the Supporting Information).^13^ This assay is based on the enzyme’s capability to bind and reduce molecular O_2_ during flavin reoxidation. Under the conditions generally used to assay MAO activity (i.e., 0.07 μM enzyme, 1.67 mM kynuramine) only the K305Q variant of human MAO A was active, whereas the other mutations appeared to totally impair enzyme functionality. The same result was obtained when the enzyme concentration was increased 25-fold. Next, we used direct spectrophotometric assays that specifically monitor the product of amine oxidation.^14^ One of these assays directly measures the absorbance of the kynuramine oxidation product at 316 nm (ε_316_ = 12 000 M^–1^ cm^–1^). Another assay is based on the tertiary amine 1-methyl-4-(1-methyl-1*H*-pyrrol-2-yl)-1,2,3,6-tetrahydropyridine (MMTP) as a substrate, which can be efficiently oxidized by MAOs with kinetic parameters only slightly lower than those of primary amines. In this protocol, the product is detected spectrophotometrically at 420 nm with a sensitivity level equivalent to that of the HRP-coupled assay.^15^ With both assays, the K305Q MAO A variant showed activity similar to that of the wild-type enzyme, whereas much lower (though detectable) activity was observed for K305M and K305S. The K305R variant was completely inactive under all conditions. We increased the enzyme concentration 25-fold to optimize the signal-to-noise ratio and measured the steady-state kinetic parameters exhibited by K305M, K305S, and K305Q MAO A mutants (Table 1). K305Q has *k*_cat_ values of the same order of magnitude as that measured for the wild-type enzyme. Instead, K305M and K305S are severely impaired in catalytic activity with *k*_cat_ values that are about 200-fold and 100-fold lower than wild-type, respectively, using both direct assays. These data suggest that replacing Lys305 with a hydrophobic side chain or with a small polar residue significantly affects MAO A catalytic efficiency, though retaining the capability to bind and oxidize the amine substrate. The longer polar side chain of Gln represents a good substitute of the conserved Lys, whereas the bulky and strongly basic Arg residue is definitely not tolerated by the enzymatic machinery. Interestingly, the *K*_m_ values are comparable for all active mutants, and only K305Q has a *K*_m_ value one order of magnitude lower than the wild-type using the MMTP assay. This observation suggests that, except for K305R, the mutations do not interfere with substrate binding within the MAO A active site.

**Table 1:** Steady-State Kinetic Parameters of MAO A Mutants Compared to Wild-Type Using MMTP and Kynuramine as Substrates

MAO A	*k*_cat_ (min^–1^)	K_m_ (mM)	*k*_cat_/*K*_m_ (min^–1^ mM^–1^)
MMTP direct assay (ε_420_ = 25000 M^–1^ cm^–1^)^a^			
wild-type	51.60 ± 1.70	0.18 ± 0.02	286.66
K305M	0.28 ± 0.01	0.36 ± 0.05	0.77
K305S	0.53 ± 0.03	0.20 ± 0.04	2.65
K305Q	21.4 ± 0.54	0.04 ± 0.005	528.50
K305R	not active	not active	not active
kynuramine direct assay (ε_316_ = 12000 M^–1^ cm^–1^)^a^			
wild-type	120.20 ± 6.70	0.15 ± 0.01	801
K305M	0.67 ± 0.02	0.18 ± 0.02	3.72
K305S	0.98 ± 0.03	0.18 ± 0.03	5.44
K305Q	91.39 ± 2.81	0.16 ± 0.02	571.18
K305R	not active	not active	not active

a All details related to the experiments are reported in the Supporting Information. Briefly, all assays were performed at 25 °C in 50 mM HEPES/NaOH at pH 7.5 containing 0.25% reduced Triton X-100 (air saturated solution). Enzyme concentration was 0.07 μM for MAO A wt and 1.8 μM for MAO A mutants.

MAOs can be irreversibly inactivated by inhibitors that form a covalent adduct with the enzyme FAD.^14^ Three classes of MAO covalent inhibitors are known that, though differing for the inactivation mechanism, all rely on the enzymatic functionality of the protein and the associated flavin cofactor. We exploited this property of MAOs to further test the Lys305 mutants. Within the propargylic class of molecules, clorgyline is a very well-known MAO A selective inhibitor that promptly forms an adduct with the flavin N5 atom that produces a sharp and intense peak at 415 nm in the UV–vis spectrum (Supporting Information Figure 1). Tranylcypromine and hydrazines are nonselective inhibitors of MAOs that behave as suicide substrates (i.e., implying a C–N bond oxidation) by reacting with either C4A or N5 atoms of the FAD cofactor, which can be detected spectrophotometrically as a bleached flavin peak. Though to different extents, both tranylcypromine and different hydrazine analogs are known to involve enzyme turnover (with molecular oxygen consumption) as part of the inactivation process; i.e., formation of the covalent adduct is preceded by unproductive cycles with a release of the oxidized inhibitor without formation of any bond with the flavin cofactor.^16^ Instead, inhibition by propargylamine compounds is not strictly dependent on the redox catalytic cycle of MAOs, being nevertheless affected by a lower functionality of the protein (as for K305M and K305S mutants). UV–vis spectral measurements showed that the K305Q can be inactivated to the same extent as the wild-type enzyme by all inhibitors, whereas inactivation of both K305M and K305S is far less effective (Supporting Information Figure 1). Furthermore, these two mutants react less efficiently with those inhibitors that require enzyme turnover such as tranylcypromine and phenelzine. The only exception is represented by phenylhydrazine that takes about 20 min to inactivate all proteins, most likely due to the lower oxygen consumption required for the inactivation process.

The experiments described above showed that the K305M mutant is impaired in enzymatic activity, but it is not completely inactive (as for K305R). MAO A catalyzes a redox reaction in which the amine substrate is oxidized with the concomitant reduction of the FAD cofactor (reductive half reaction; Figure 1). The catalytic turnover is guaranteed by the prompt flavin reoxidation by molecular O_2_ (oxidative half reaction).^4,17^ To better dissect the role of Lys305 in H_2_O_2_ production by MAO A, we monitored the K305M enzyme reaction spectrophotometrically under anaerobiosis conditions, which were essential to keep the enzyme in the reduced state as the mutant is likely to retain some O_2_ reactivity. This specific mutant (rather than K305S) was selected for these further studies because its side chain structurally mimics that of lysine, though returning an impaired turnover efficiency due to the lack of the positive charge that prevents an optimal interaction with the flavin through water as observed in wild-type MAO A. Incubation of the oxygen-free enzyme sample with a tyramine substrate led to full reduction of the flavin coenzyme, which could be stably reached with both wild-type and the K305M mutant in a few minutes (Figure 2A). When aerobic conditions were restored by exposing the system to air, the wild-type enzyme was promptly reoxidized, whereas in the case of K305M the reappearance of the oxidized flavin peak was only gradually obtained and required more than 1 h to reach completion. In agreement with the steady-state kinetic parameters (Table 1), these results indicate that K305M can bind and oxidize the amine substrate like the wild-type enzyme, while it is heavily impaired in oxidized flavin regeneration by molecular oxygen.

**Figure 2:**
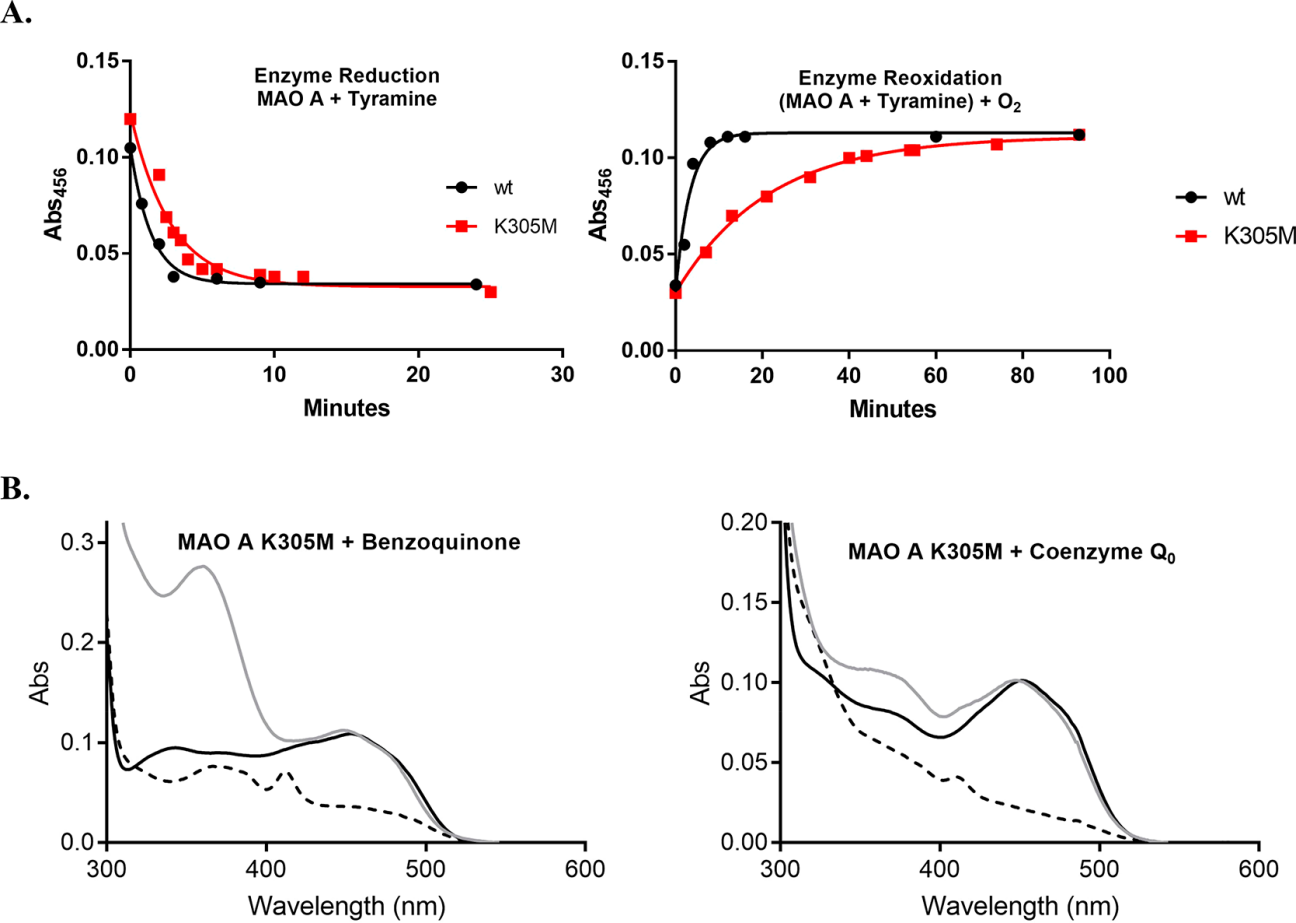
Spectrophotometric measurements of MAO A activity under anaerobiosis conditions. In all experiments, the cuvette contained 10 μM enzyme in 50 mM potassium phosphate at pH 7.8, 300 mM sodium chloride, 20% (w/v) glycerol, and 0.05% (w/v) Fos-Choline-12. (A) Flavin reduction was obtained by anaerobically adding 1 mM tyramine; reoxidation by molecular oxygen was monitored for the K305M mutant (red) compared to the wild-type enzyme (black). Enzyme reduction (left panel) was followed by measuring the absorbance at 456 nm corresponding to the peak of the oxidized flavin spectrum, which is bleached when flavin is reduced by the amine substrate. Enzyme reoxidation was monitored through the reappearance of the peak centered at 450 nm after exposure of the reaction mix to oxygen. Supporting Information Figure 2A and B show the overall UV–vis spectra of the oxidized and reduced enzyme for wild-type and K305M, respectively. (B) K305M flavin reoxidation by alternative electron acceptors: 200 μM benzoquinone (left) and 50 μM coenzyme Q_0_ (right). UV–vis spectra of the oxidized (initial), photoreduced, and reoxidized K305M mutant are depicted as continuous black, dashed black, and gray lines, respectively. In this experiment, photochemical reduction of the enzyme was preferred to avoid multiple turnovers. In both panels, the profile of the photoreduced enzyme is consistent with a mixture of the anionic semiquinone and hydroquinone flavin forms that were previously observed for MAOs.^18^ The peak at 350 nm in the left panel is due to benzoquinone absorbance.

Next, we asked if the K305M mutant could be reoxidized by electron acceptors alternative to molecular oxygen. Although MAOs are “true” oxidases, their active site might bind organic molecules that accept electrons and, in the case of the K305M mutant, may work more efficiently than oxygen. Among these, quinones are electrophilic compounds normally found in mitochondria and other organelles where they serve as acceptors in cellular electron transport chains. It was reported that some quinones and other redox compounds display binding affinity for MAO A.^19,20^ We tested benzoquinone and coenzyme Q_0_ for their ability to reoxidize the K305M mutant under anaerobiosis conditions (Figure 2B). The former is the basic unit of quinones, the latter is the reactive part of the naturally occurring coenzyme Q_10_ that is highly hydrophobic and therefore very difficult to experimentally handle in acqueous solutions. For this experiment, K305M MAO A was anaerobically photoreduced rather than incubated with substrate to avoid any direct electron transfer from the substrate to the electron acceptor or multiple turnovers. The addition of either benzoquinone or coenzyme Q_0_ readily led to the full reappearance of the peak at 456 nm, indicating that these molecules can reoxidize the enzyme-bound FAD coenzyme. The same experiment was carried out also with the wild-type enzyme using benzoquinone (Supporting InformationFigure 3) obtaining similar results. Benzoquinone and coenzyme Q_0_ were also tested for their ability to restore the normal enzyme turnover under aerobic conditions using the assays described above, but no significant change in enzymatic activity of K305M was observed. This may be due to the fact that the mutant is endowed with a, though limited, reactivity with oxygen and that the large amount of substrate may hamper the accessibility of the alternative electron acceptor. These results outlined the K305M MAO A mutant as an oxygen-inert tool to be tested in a cellular model of cardiomyocytes where the bioavailability of quinone-based electron acceptors may restore an efficient enzyme regeneration.

**Figure 3:**
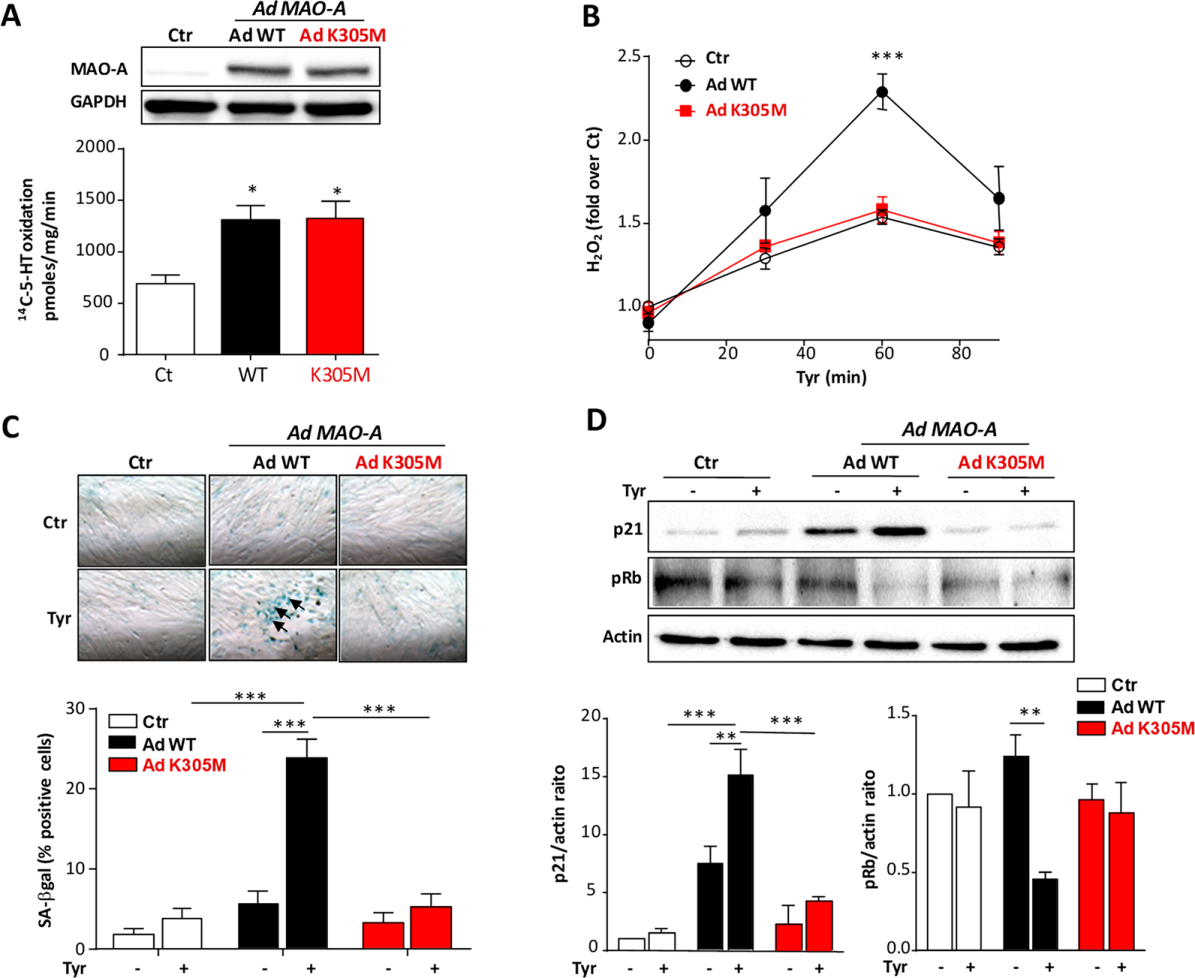
Cellular effects of wild-type and K305M mutant in H9C2 cells. Cells were transduced or not (Ctr) with adenovirus carrying either the wild-type (Ad WT) or mutant K305M (Ad K305M) MAO A. Assays were performed 72 h post-transduction. (A) MAO A expression was measured by immunoblot (upper panel, *n* = 4), and MAO A activity was determined by radioactive assay (lower panel, *n* = 4). (B) H_2_O_2_ was measured with Amplex Red assay in extracellular media at the times indicated after tyramine (500 μM) exposure (*n* = 3). (C) For SA-βgal activity (*n* = 4) and (D) immunoblots of p21 and phospho-Rb (*n* = 3), cells were preincubated 4 h with clorgyline before adenoviral transduction to block endogenous MAO A activity. After 72 h of tyramine treatment (500 μM), cells were monitored for senescence markers. SA-βgal activity was represented as % of blue cells (arrows). Data are expressed as means ± SEM. ***p < 0.001, **p < 0.01, *p < 0.05 vs indicated value.

We thereby generated two adenoviral constructs in order to overexpress wild-type (Ad WT) and K305M (Ad K305M) human MAO A in cardiomyoblasts. As shown in Figure 3A, MAO A protein levels were similarly increased in cells transduced with Ad WT or Ad K305M, compared to control untransduced cells (Ctr). Interestingly, MAO A activity, measured as the quantity of products formed by ^14^C-5-HT degradation, was equally increased in Ad WT and Ad K305M-transduced cells compared to Ctr. Thus, K305M appears to have turnover activity similarly to wild-type when expressed in cells, which raised the question whether the mutant uses O_2_ or an alternative electron acceptor in cells. We directly measured the concentration of H_2_O_2_ generated during the oxidation of tyramine. As shown in Figure 3B, tyramine addition led to H_2_O_2_ production in Ctr cells with a maximum at 60 min, corresponding to endogenous MAO A activation. Most interestingly, Ad WT transduction potentiated H_2_O_2_ production upon tyramine administration, which was instead not observed for Ad K305M. This demonstrates that K305M mutant is able to degrade MAO substrate without production of H_2_O_2_. Thus, we took advantage of this unique property to establish the contribution of ROS in the senescent response induced by MAO A.^8^ To better establish the specific effects of wild-type and K305M MAO A in cardiomyoblasts, we blocked endogenous MAO A with the irreversible inhibitor clorgyline before performing adenoviral transduction and treatment with tyramine (Supporting Information Figure 4A). In cells transduced with Ad WT, but not K305M, tyramine application for 30 min significantly increased ROS production compared to Ctr (Supporting Information Figure 4). We next evaluated the chronic effect of MAO A stimulation over 3 days with tyramine on aging markers by measuring senescence-associated-βgal (SA-βgal), a β-galactosidase activity detectable at pH 6.0 in senescent cells. Interestingly, SA-βgal activity was increased only in cells expressing Ad WT but not K305M. Similarly, the senescence marker p21 accumulated in Ad WT cells but not in Ad K305M cells, while the retinoblastoma protein (Rb) was dephosphorylated only in tyramine-activated Ad WT cells, preventing the progression of the cell cycle (Figure 3D). Altogether, our results show that substituting Lys305 with Met in MAO A impairs the production of H_2_O_2_, preventing oxidative stress and senescence induced by a chronic activation of MAO A.

In conclusion, our work provided an insightful investigation on the role of the conserved lysine residue of flavin-dependent amine oxidases in enzyme turnover and on the effects of H_2_O_2_ generated by human MAO A in cardiac cell aging. We demonstrated that in human MAO A, the K305M mutant retains the capability to bind and oxidize the amine substrate, while it is significantly impaired in reoxidation by molecular O_2_. Using this mutant as a mimic of an oxygen-inert enzyme in the context of cardiomyocyte cells, we gave further support to the hypothesis that, in the heart, MAO A represents a noteworthy source of ROS promoting cell senescence. From this perspective, pharmacological treatments targeting MAO A, so far limited to neurological diseases, may be extended to prevent heart failure in some frailty conditions of aged patients. Moreover, since many reports indicate that MAO A promotes prostate and glioblastoma tumorigenesis and metastasis, this approach may also limit cancer growth.^21^

## Supporting Information Available

Details of the experimental methods, supporting Table 1, supporting Figures 1–4 (PDF)

## References

[ref1] Sies H.; Berndt C.; Jones D. P. (2017) Oxidative Stress. Annu. Rev. Biochem. 86, 715–748. 10.1146/annurev-biochem-061516-045037. 28441057

[ref2] Sies H. (2014) Role of metabolic H_2_O_2_ generation: redox signaling and oxidative stress. J. Biol. Chem. 289, 8735–8741. 10.1074/jbc.R113.544635. 24515117 PMC3979367

[ref3] Iacovino L. G.; Magnani F.; Binda C. (2018) The structure of monoamine oxidases: past, present, and future. J. Neural. Transm. (Vienna) 125, 1567–1579. 10.1007/s00702-018-1915-z. 30167931

[ref4] Romero E.; Gómez Castellanos J. R.; Gadda G.; Fraaije M. W.; Mattevi A. (2018) Same Substrate, Many Reactions: Oxygen Activation in Flavoenzymes. Chem. Rev. 118, 1742–1769. 10.1021/acs.chemrev.7b00650. 29323892

[ref5] Youdim M. B.; Edmondson D. E.; Tipton K. F. (2006) The therapeutic potential of monoamine oxidase inhibitors. Nat. Rev. Neurosci. 7, 295–309. 10.1038/nrn1883. 16552415

[ref6] Maurel A.; Hernandez C.; Kunduzova O.; Bompart G.; Cambon C.; Parini A.; Francés B. (2003) Age-dependent increase in hydrogen peroxide production by cardiac monoamine oxidase A in rats. Am. J. Physiol. Heart Circ. Physiol. 284, H1460–1467. 10.1152/ajpheart.00700.2002. 12531732

[ref7] Villeneuve C.; Guilbeau-Frugier C.; Sicard P.; Lairez O.; Ordener C.; Duparc T.; De Paulis D.; Couderc B.; Spreux-Varoquaux O.; Tortosa F.; Garnier A.; Knauf C.; Valet P.; Borchi E.; Nediani C.; Gharib A.; Ovize M.; Delisle M. B.; Parini A.; Mialet-Perez J. (2013) p53-PGC-1α pathway mediates oxidative mitochondrial damage and cardiomyocyte necrosis induced by monoamine oxidase-A upregulation: role in chronic left ventricular dysfunction in mice. Antioxid. Redox Signaling 18, 5–18. 10.1089/ars.2011.4373. PMC3503466 22738191

[ref8] Manzella N.; Santin Y.; Maggiorani D.; Martini H.; Douin-Echinard V.; Passos J. F.; Lezoualc’h F.; Binda C.; Parini A.; Mialet-Perez J. (2018) Monoamine oxidase-A is a novel driver of stress-induced premature senescence through inhibition of parkin-mediated mitophagy. Aging Cell 17, e12811 10.1111/acel.12811. 30003648 PMC6156293

[ref9] McDonald C. A.; Fagan R. L.; Collard F.; Monnier V. M.; Palfey B. A. (2011) Oxygen reactivity in flavoenzymes: context matters. J. Am. Chem. Soc. 133, 16809–16811. 10.1021/ja2081873. 21958058 PMC3203534

[ref10] Henderson Pozzi M.; Fitzpatrick P. F. (2010) A lysine conserved in the monoamine oxidase family is involved in oxidation of the reduced flavin in mouse polyamine oxidase. Arch. Biochem. Biophys. 498, 83–88. 10.1016/j.abb.2010.04.015. 20417173 PMC2880204

[ref11] Zhao G.; Bruckner R. C.; Jorns M. S. (2008) Identification of the oxygen activation site in monomeric sarcosine oxidase: role of Lys265 in catalysis. Biochemistry 47, 9124–9135. 10.1021/bi8008642. 18693755 PMC2764408

[ref12] Li M.; Hubálek F.; Newton-Vinson P.; Edmondson D. E. (2002) High-level expression of human liver monoamine oxidase A in *Pichia pastoris*: comparison with the enzyme expressed in Saccharomyces cerevisiae. Protein Expression Purif. 24, 152–162. 10.1006/prep.2001.1546. 11812236

[ref13] Veitch N. C. (2004) Horseradish peroxidase: a modern view of a classic enzyme. Phytochemistry 65, 249–59. 10.1016/j.phytochem.2003.10.022. 14751298

[ref14] Ramsay R. R.; Albreht A. (2018) Kinetics, mechanism, and inhibition of monoamine oxidase. J. Neural. Transm. (Vienna) 125, 1659–1683. 10.1007/s00702-018-1861-9. 29516165

[ref15] Bissel P.; Bigley M. C.; Castagnoli K.; Castagnoli N. Jr. (2002) Synthesis and biological evaluation of MAO-A selective 1,4-disubstituted-1,2,3,6-tetrahydropyridinyl substrates. Bioorg. Med. Chem. 10, 3031–3041. 10.1016/S0968-0896(02)00136-0. 12110326

[ref16] Binda C.; Wang J.; Li M.; Hubalek F.; Mattevi A.; Edmondson D. E. (2008) Structural and mechanistic studies of arylalkylhydrazine inhibition of human monoamine oxidases A and B. Biochemistry 47, 5616–5625. 10.1021/bi8002814. 18426226

[ref17] Chaiyen P.; Fraaije M. W.; Mattevi A. (2012) The enigmatic reaction of flavins with oxygen. Trends Biochem. Sci. 37, 373–380. 10.1016/j.tibs.2012.06.005. 22819837

[ref18] Arslan B. K.; Edmondson D. E. (2010) Expression of zebrafish (*Danio rerio*) monoamine oxidase (MAO) in *Pichia pastoris*: purification and comparison with human MAO A and MAO B. Protein Expression Purif. 70, 290–297. 10.1016/j.pep.2010.01.005. PMC2827670 19883764

[ref19] Ramsay R. R.; Dunford C.; Gillman P. K. (2007) Methylene blue and serotonin toxicity: inhibition of monoamine oxidase A (MAO A) confirms a theoretical prediction. Br. J. Pharmacol. 152, 946–951. 10.1038/sj.bjp.0707430. 17721552 PMC2078225

[ref20] Paudel P.; Seong S. H.; Shrestha S.; Jung H. A.; Choi J. S. (2019) In Vitro and in Silico Human Monoamine Oxidase Inhibitory Potential of Anthraquinones, Naphthopyrones, and Naphthalenic Lactones from Cassia obtusifolia Linn Seeds. ACS Omega. 4, 16139–16152. 10.1021/acsomega.9b02328. 31592482 PMC6777294

[ref21] Shih J. C. (2018) Monoamine oxidase isoenzymes: genes, functions and targets for behavior and cancer therapy. J. Neural Transm (Vienna) 125, 1553–1566. 10.1007/s00702-018-1927-8. 30259128 PMC6245662

