# Supplementary material for: Rational Redesign of Monoamine Oxidase A into a Dehydrogenase to Probe ROS in Cardiac Aging
Source: ACS Chem Biol. 2020 Jun 26;15(7):1795–800. doi: 10.1021/acschembio.0c00366 (PMC8009472; doi:10.1021/acschembio.0c00366)
Supplement: Supplementary file 1 — cb0c00366_si_001.pdf [file cb0c00366_si_001.pdf]

Supporting Information

Rational re-design of Monoamine Oxidase A into a dehydrogenase to probe ROS in cardiac ageing

Luca Giacinto Iacovino[†], Nicola Manzella[§], Jessica Resta[§], Maria Antonietta Vanoni[¶], Laura Rotilio[†], Leonardo Pisani[#], Dale Edward Edmondson[¥], Angelo Parini[§], Andrea Mattevi[†], Jeanne Mialet-Perez[§], Claudia Binda[†,*]

[†]Department of Biology and Biotechnology, University of Pavia, Milan, Italy

[§]Institute of Metabolic and Cardiovascular Diseases (I2MC), Institut National de la Santé et de la Recherche Médicale (INSERM), Université de Toulouse, Toulouse, France

[¶]Department of Biosciences, University of Milan, Milan, Italy

[#]Department of Pharmacy-Drug Sciences, University of Bari Aldo Moro, Bari, Italy

[¥]Department of Biochemistry, Emory University, Atlanta, GA, USA

* Corresponding author: Claudia Binda

E-mail: claudia.binda@unipv.it. Phone: (+39)0382-985527

ORCID: 0000-0003-2038-9845

Index

Methods	page 3
Supporting Table 1	page 7
Supporting Information Figure 1	page 8
Supporting Information Figure 2	page 9
Supporting Information Figure 3	page 10
Supporting Information Figure 4	page 11
References	page 12

Methods

Reagents. MMTP (1-methyl-4-(1-methyl-1H-pyrrol-2-yl)-1,2,3,6-tetrahydropyridine) oxalate salt was synthetized as previously described by Jian Yu and Castagnoli.[1] All other reagents were purchased from Sigma-Aldrich except for detergents that were from Anatrace.

MAO A mutants expression and purification. A DNA fragment encoding the full-length human MAO A was inserted into a pPIC 3.5 vector (Invitrogen) and used as template for site-specific mutagenesis. The primers are listed in Supporting Table 1 and correct incorporation of the mutation was checked by outsource sequencing (Eurofins Genomics). The mutant MAO A plasmids were linearized with MssI and transformation in *Pichia pastoris* was carried out by electroporation using KM71 strain (Invitrogen). The His^+ transformants were selected on minimal media plates without histidine, followed by random selection of eight colonies for small-scale expression analysis. The transformants yielding the highest level of protein expression were chosen for scale-up coltures. MAO A wild type and mutants were purified as previously described with few modifications of the published protocols.[2] Briefly, 30 g cells were resuspended in 100 ml of breaking buffer (50 mM sodium phosphate pH 7.2, 5 % (w/v) glycerol, 1 mM PSMF, 30 µM DTT, 1 mM EDTA) with an equal volume of silica-zirconia beads (0.5 mm in diameter) and then disrupted in a Beadbeater (Hamilton Beach Blender 908). The cell lysate was separated from beads through filtration with a layer of Miracloth (Calbiochem) followed by low speed centrifugation (1500 x g for 10 minutes at 4°C). The supernatant was centrifuged at high speed (70000 x g for 40 minutes at 4°C) in order to separate the membrane fraction. The pellet was resuspended to a final concentration of 15 mg/ml in 50 mM sodium phosphate pH 7.8, 300 mM sodium chloride, 20 mM imidazole, 20 % (w/v) glycerol, 1 mM PMSF, 30 µM DTT. The protein concentration was determined using the Biuret method.[3] Fos-Choline-12 was added to a concentration of 1 % (w/v) and the mixture was stirred at 4°C in the dark for 1 hour. After centrifugation (70000 x g for 30 minutes) the extract was loaded onto a 5 ml HisTrap column (GE-Healthcare) equilibrated with the buffer used for the resuspension. After washing with 10 column volumes of the buffer, the enzyme was eluted with 50 mM sodium phosphate buffer pH 7.8, 300 mM sodium chloride, 300 mM imidazole, 20 % glycerol (w/v), 1 mM PMSF, 30 µM DTT. The enzyme fractions were pooled according to the ratio of the absorbance at 280 nm and 456 nm, and concentrated with Amicon Ultra 30K centrifugal filters (Millipore). The excess of imidazole was removed with a 5 ml HiTrap Desalting column (GE Healthcare) equilibrated with 50 mM sodium phosphate buffer pH 7.8, 300 mM sodium chloride, 20 % (w/v) glycerol. The purity of the protein was determined by SDS-PAGE analysis and inspection of the UV-Vis spectrum. The final concentration of the pure protein was obtained spectrophotometrically with a NanoDrop ND-1408

1000 (Thermo Fisher Scientific Inc.) from the absorbance of the enzyme-bound FAD coenzyme at 456 nm (ε_{456} = 12000 M^{-1} cm^{-1}).

Steady-state kinetic analyses. The enzymatic activity of all MAO A constructs was tested by spectrophotometric measurements in the presence of different concentrations of kynuramine and MMTP as substrates using a Cary 100 UV/Vis spectrophotometer. In each assay the initial velocity value (v_0) was calculated from the absorbance changes at selected wavelengths and the known extinction coefficients. All experiments were performed at 25 °C in 50 mM Hepes/NaOH pH 7.5 containing 0.25 % (v/v) reduced Triton X-100. The rate of MMTP oxidation was monitored spectrophotometrically at 420 nm (ε_{420} = 25000 M^{-1} cm^{-1}),[4] whereas kynuramine consumption was detected at 316 nm (ε_{316} = 12000 M^{-1} cm^{-1}) in the direct assay[5] or at 515 nm when using the horseradish peroxidase (HRP) coupled assay that monitors the production of hydrogen peroxide.[6] In the direct assays the reaction was started by adding the enzyme at a final concentration of 0.07 µM for MAO A wt and 1.8 µM for MAO A mutants. In the HRP-coupled assay, the assay mixture contained 0.1 mM 4-aminoantipyrine, 1 mM 3,5-dichloro-2-hydroxybenzenesulfonic acid, 0.35 µM HRP and varying concentrations of kynuramine. The reaction was started by adding the enzyme at a final concentration of 0.07 µM for MAO A wt and 10 µM for mutants. Data were fit to the Michaelis-Menten equation $v_0 = v_{max}[S]/(K_m + [S])$ with GraphPad 5.0 software that provides the parameter values and the associated errors.

Covalent inhibition analyses. Irreversible inhibition of MAO A wt and mutants was tested by UV-Vis spectral measurement (Supporting Figure 1) using an Agilent 8453 UV/Vis diode-array spectrophotometer. The experiments were performed in presence of 10 µM of enzyme with 10-fold molar excess of irreversible inhibitor. The buffer used was 50 mM potassium phosphate pH 7.8, 300 mM sodium chloride, 20 % (w/v) glycerol, 0.05 % (w/v) Fos-Choline-12 in 150 µL final volume at 25°C. The reaction was monitored at different times until no further modification was detected. The final spectra and time-course of absorbance changes observed with each inhibitor were plotted using GraphPad 5.0 software.

Anaerobiosis experiments. All UV/Vis spectra analyses were performed using an Agilent HP 8453 spectrophotometer with a home-made quartz cuvette equipped with side-arms and connected with a vacuum/nitrogen apparatus.[7] Anaerobic solutions of 10 µM enzyme in 50 mM potassium phosphate pH 7.8, 300 mM sodium chloride, 20 % (w/v) glycerol, 0.05 % (w/v) Fos-Choline-12 were prepared in the tonometer 1 mL cuvette by repeating 50 cycles of evacuation and equilibration with oxygen-

free nitrogen. The buffer was the same as that used for aerobic experiments. Tyramine was used as substrate in 100-fold molar excess with respect to enzyme, made anaerobic separately in the side arm of the tonometer and anaerobically added to the enzyme solution to start the reaction. The re-oxidation of the enzyme was carried out by opening the cuvette and mixing the solution in order to promote the re-solubilization of oxygen. The time-course of FAD reduction/oxidation was monitored at the maximum of absorption at 456 nm (ε_{456} = 12000 M^{-1} cm^{-1}). In photoreduction experiments 10 µM enzyme was in the 1 ml quartz cuvette of the tonomenter mixed in the presence of 1 µM of deazaflavin and 10 mM EDTA[8] whereas the electron acceptors (either 200 µM p-benzoquinone or 50 µM coenzyme Q_0) were placed into the side arm of the anaerobic apparatus, and the system was made anaerobic as previously described. The anaerobic cuvette was placed in a water bath and illuminated with a projector lamp. After each period of irradiation (0.5 – 2 minutes) the absorbance spectrum was recorded until no spectral changes were observed. Once the flavin was stably reduced, the selected electron acceptor was added to the reduced enzyme to monitor the re-oxidation by acquiring spectra at different times.

Generation of adenoviral constructs. Adenovirus were generated using the RapaCMV adenoviral expression system (Cell Biolabs) according to manufacturer’s instructions. Briefly, cDNAs of WT or K305M human MAO A were excised from PCR8 plasmid and inserted into the shuttle vector exploiting the *BamH*I and *Not*I restriction sites. Adenovirus were amplified in HEK293 cells and purified by cesium chloride gradient.

Cell culture and treatments. Rat H9c2 cardiomyoblasts obtained from the American Type Culture Collection (Rockville, MD, U.S.A.) were grown in Dulbecco's Modified Eagle Medium (DMEM, Thermo Fisher) containing 10% heat-inactivated fetal bovine serum (FBS) under 5% CO_2 at 37°C.

MAO A activity. Frozen pellets of H9C2 cells were homogenized in 50 mM sodium phosphate pH 7.5, supplemented with protease inhibitors and sonicated. Crude extracts (40 µg) were incubated at 37°C for 30 min, in the presence of 400 µM of [^{14}C]-serotonin. To define non specific activities, MAO A inhibitor clorgyline was used (0.1 µM). The reaction was stopped by the addition of 0.1 ml of HCl 4N at 4°C. The reaction product was extracted (efficiency 92%) with 1 mL of ethyl acetate/toluene (v/v), and the radioactivity contained in the organic phase was counted by liquid scintillation.

Western blot. H9C2 cells were lysed in 0.150 ml RIPA buffer (20 mM Tris-HCl pH 7.5, 150 mM NaCl, 1 mM EDTA, 1% (v/v) NP40, 0.1% (v/v) SDS, antiphosphates and antiproteases). Following centrifugation at 13,000 g for 5 min, the supernatant was collected and equal amount of proteins were subjected to SDS-PAGE electrophoresis and transferred to a nitrocellulose membrane (Trans-blot turbo transfer system, Bio-Rad). Primary antibody incubations were performed overnight with 1:1000 dilutions for anti-MAO A, anti-phospho-Rb (Cell Signaling) and anti-p21 (Santa Cruz Biotechnology) IgG followed by incubation with secondary antibodies for 1 h at room temperature (1:5000 dilutions). ECL (Life sciences) was used for detection and images were recorded with the BioRad ChemiDoc MP, Imaging System. Relative densities were quantified using Image-Lab 4.0 software (Bio-Rad).

ROS production. Extracellular H_2O_2 detection was evaluated with Amplex-Red probe (Invitrogen, Molecular Probes). Briefly, cells were seaded in 96-wells, treated with tyramine (500 µM) and the supernatant collected and mixed with a Amplex red assay solution for 30 min. Total cellular ROS were measured using the fluorescent probe DCFDA assay at a concentration of 5 µM (Thermo Fisher Scientific).

SA-βgal staining. SA-βGal activity was measured as previously described.[9] Briefly, cells were fixed with paraformaldehyde for 10 minutes, washed with PBS buffer (137 mM NaCl, 2.7 mM KCl, 8 mM Na_2HPO_4, and 2 mM KH_2PO_4), and stained with SA-β-Gal stain solution (1 mg/ml X-gal dissolved in DMSO, 40 mM citric acid/sodium phosphate buffer pH 6.0, 5 mM potassium ferrocyanide, 5 mM potassium ferricyanide, 150 mM NaCl, 2mM $MgCl_2$). After 16 hours, images were taken using a bright-fieldmicroscope.

Statistical analysis. Statistical analysis of significance was calculated using t-Student test, 1-way or 2-way ANOVA when appropriate using GraphPad Prism 6 (GraphPad Software, La Jolla, CA, USA). The results were shown as the means ± SEM. Values of p= 0.05, 0.01, and 0.001 were considered as statistically significant.

Supporting Table 1. Primers utilized for recombinant human MAO A mutagenesis

K305M	TCATTATGTGCATGATGTATTACAAG
K305S	AGCTGTCATTTCTTGCATGATGTATTACAAG
K305Q	GCTGTCATTCAATGCATGATGTATTACAAG
K305R	AGCTGTCATTCGCTGCATGATGTATTACAA

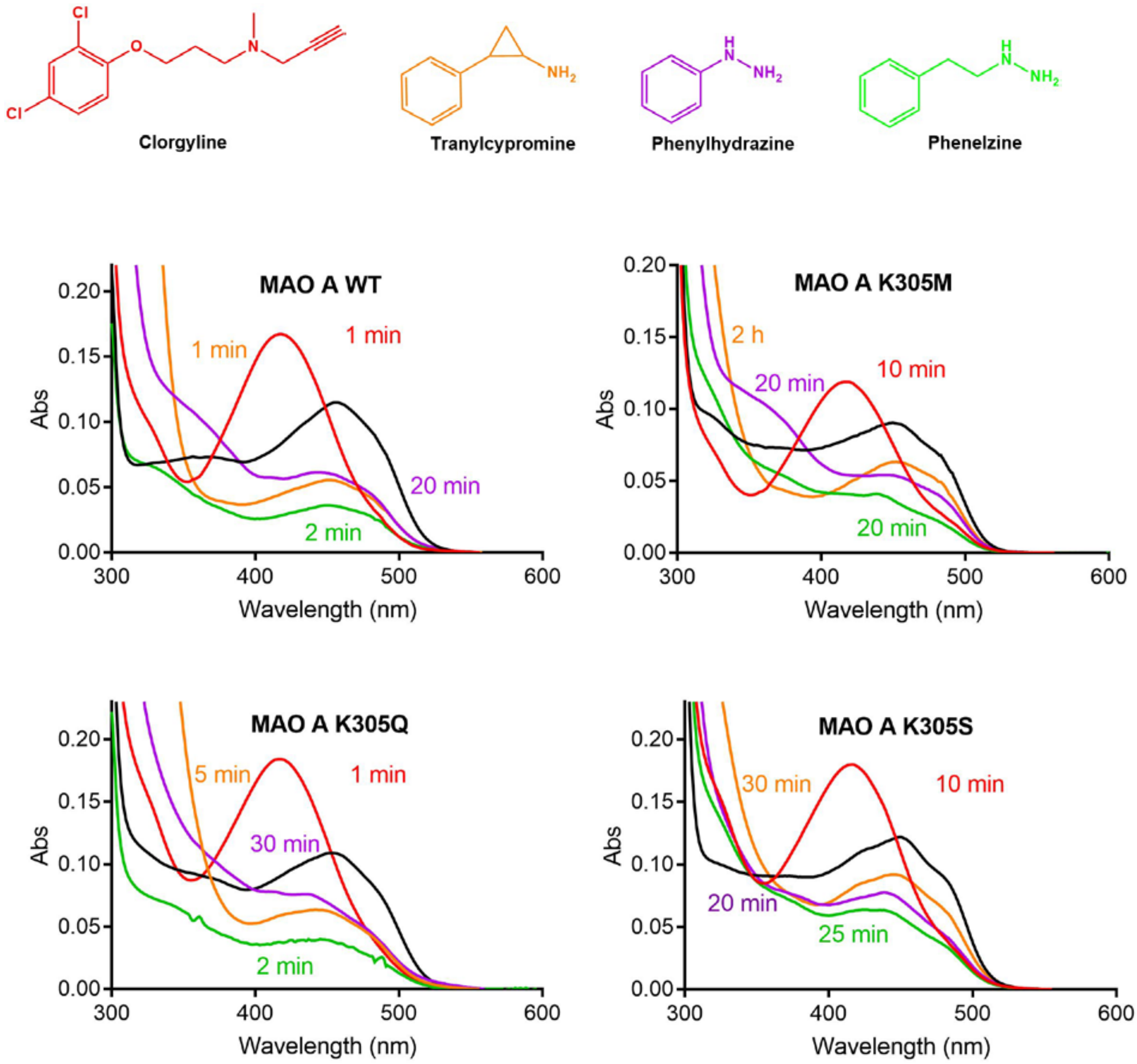

Supporting Figure 1. UV-Vis spectra of human MAO A (wild type enzyme and active mutants) during the time-dependent inactivation by covalent inhibitors. The absorption spectrum of the oxidized protein (prior to inhibitor additon) is in black, whereas the red, green, orange and purple traces indicate the enzyme inactivated by clorgyline, phenelzine, tranylcypromine and phenylhydrazine, respectively. Time required to reach the final spectrum is also specified (1 minute is the minimum recorded time after addition of the inhibitor to the enzyme solution, i.e. it may imply an even faster inactivation process). K305R MAO A does not react with any covalent inhibitor (data not shown).

A.

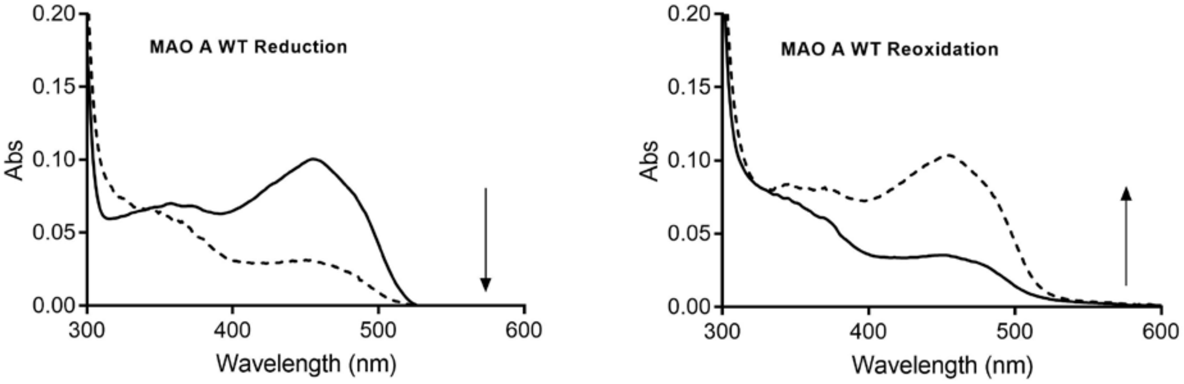

B.

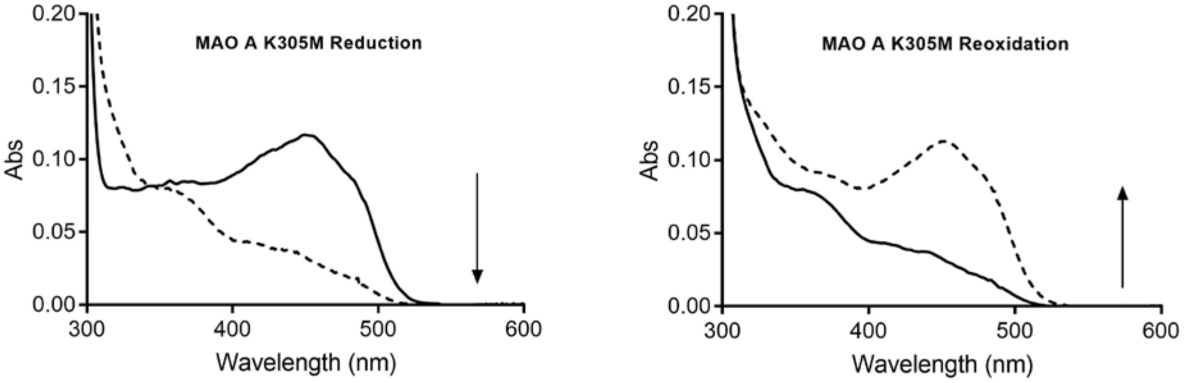

Supporting Figure 2. UV-vis spectra of wild type (A) and K305M (B) MAO A flavin reduction under anaerobiosis and reoxidation by molecular oxygen. In all panels the initial and final spectra are represented by continuous and dashed lines, respectively. The arrow indicates the bleaching of the flavin absorption peak centered at 456 nm in the reductive half-reaction and the reappearance of the peak during the oxidative half-reaction.

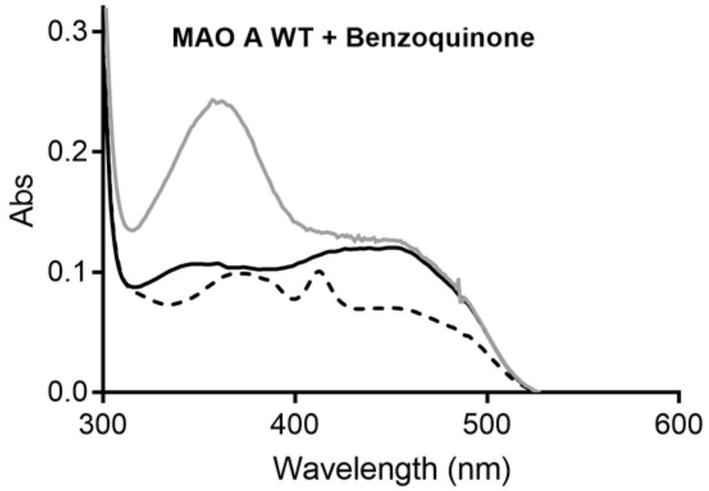

Supporting Figure 3. Spectrophotometric measurements of wild type MAO A flavin reoxidation by benzoquinone as alternative electron acceptor. UV-Vis spectra of the oxidized (initial), photoreduced and reoxidized MAO A are depicted as continuous black, dashed black and gray lines, respectively.

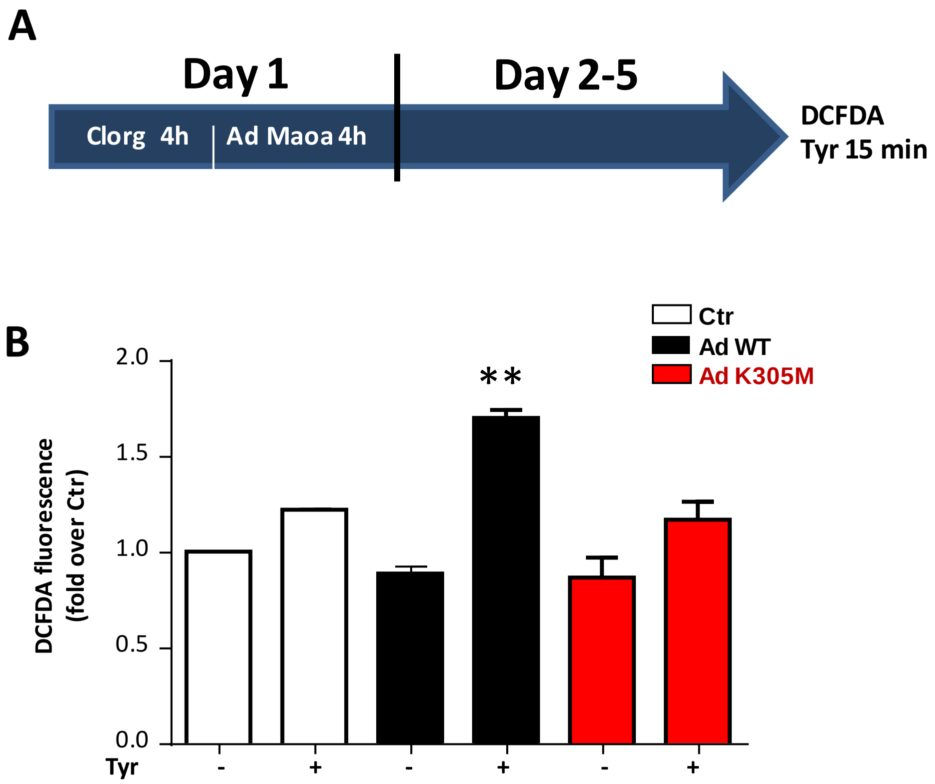

Supporting Figure 4. Total cellular ROS were measured with the DCFDA probe in H9C2 cells transduced with WT or K305M mutant. (A) Cells were preincubated for 4 h with clorgyline to block endogenous MAO A activity, before performing adenoviral transduction. (B) 72 h post-transduction, fluorescence of DCFDA was measured in the presence of 500 µM tyramine (Tyr in the figure) for 30 min. Data are expressed as means ± sem.

References

1. Yu, J., and Castagnoli, N. (1999) Synthesis and MAO-B Substrate Properties of 1-Methyl-4-heteroaryl-1,2,3,6-tetrahydropyridines. *Bioorganic & Medicinal Chemistry 7,* 231-239.

2. Li, M., Hubálek, F., Newton-Vinson, P., and Edmondson D. (2002) High-Level Expression of Human Liver Monoamine Oxidase A in Pichia pastoris: Comparison with the Enzyme Expressed in Saccharomyces cerevisiae. *Protein Expression and Purification. 24,* 152-162.

3. Layne, E. (1957) Spectrophotometric and turbidimetric methods for measuring proteins. *Methods in Enzymology 3,* 447-454.

4. Bissel, P., Bigley, M., Castagnoli, K., and Castagnoli, Jr. N. (2002) Synthesis and Biological Evaluation of MAO-A Selective 1,4-Disubstituted-1,2,3,6-tetrahydropyridinyl Substrates. *Bioorganic & Medicinal Chemistry 10,* 3031-3041.

5. Weissbach, H., Smith, T.E., Daly, J.W., Witkop, B., Udenfriend, S. (1960) A rapid spectrophotometric assay of mono-amine oxidase based on the rate of disappearance of kynuramine. *J. Biol. Chem. 235,* 1160-1163.

6. Vojinović, V., Azevedo A.M., Martins, V.C.B., Cabral, J., Gibson, T.D., and Fonseca, L.P. (2004) Assay of H_2O_2 by HRP catalysed co-oxidation of phenol-4-sulphonic acid and 4-aminoantipyrine: characterisation and optimisation. *Journal of Molecular Catalysis B: Enzymatic 28,* 129-135.

7. Williams, C.H. Jr., Arscott, L.D., Matthews, R.G., Thorpe, C., and Wilkinson, K.D. (1979) Methodology employed for anaerobic spectrophotometric titrations and for computer-assisted data analysis. *Methods Enzymol. 62,* 185-198.

8. Massey, V., and Hemmerich, P. (1978) Photoreduction of flavoproteins and other biological compounds catalyzed by deazaflavins. Biochemistry 17, 9-16.

9. Manzella, N., Santin, Y., Maggiorani, D., Martini, H., Douin-Echinard, V., Passos, J.F., Lezoualc'h, F., Binda, C., Parini, A., and Mialet-Perez, J. (2018) Monoamine oxidase-A is a novel driver of stress-induced premature senescence through inhibition of parkin-mediated mitophagy. *Aging Ce*ll 17, e12811.
